# Phosphorylated CAV1 activates autophagy through an interaction with BECN1 under oxidative stress

**DOI:** 10.1038/cddis.2017.71

**Published:** 2017-05-25

**Authors:** Jihoon Nah, Seung-Min Yoo, Sunmin Jung, Eun Il Jeong, Moonju Park, Bong-Kiun Kaang, Yong-Keun Jung

**Affiliations:** 1 Department of Biological Sciences, Seoul National University, 1 Gwanak-ro, Seoul, Gwanak-gu 151-747, Korea

## Abstract

CAV1/Caveolin1, an integral membrane protein, is involved in caveolae function and cellular signaling pathways. Here, we report that CAV1 is a positive regulator of autophagy under oxidative stress and cerebral ischemic injury. Treatment with hydrogen peroxide enhanced autophagy flux and caused the localization of BECN1 to the mitochondria, whereas these changes were impaired in the absence of CAV1. Among many autophagy signals, only LC3 foci formation in response to hydrogen peroxide was abolished by CAV1 deficiency. Under oxidative stress, CAV1 interacted with a complex of BECN1/VPS34 through its scaffolding domain, and this interaction facilitated autophagosome formation. Interestingly, the phosphorylation of CAV1 at tyrosine-14 was essential for the interaction with BECN1 and their localization to the mitochondria, and the activation of autophagy in response to hydrogen peroxide. In addition, the expression of a phosphatase PTPN1 reduced the phosphorylation of CAV1 and inhibited autophagy. Further, compared to that in wild-type mice, autophagy was impaired and cerebral infarct damage was aggravated in the brain of *Cav1* knockout mice. These results suggest that the phosphorylated CAV1 functions to activate autophagy through binding to the BECN1/VPS34 complex under oxidative stress and to protect against ischemic damage.

Autophagy is a major protective pathway that is activated in cells to adapt to various stresses. In this process, double-membrane vesicles known as autophagosomes engulf proteins or organelles to maintain homeostasis.^1, 2^ In recently years, evidence has been found of a cytoprotective role for autophagy in various diseases, including neurodegeneration and ischemic injury.^3^ Autophagy is elaborately controlled by several intracellular signaling pathways and through multiple autophagy-related (ATG) proteins. Among over 35 ATG genes, BECN1 plays the key role in nucleating autophagic vacuoles and is a component of the BECN1/VPS34/VPS15 complex (also known as phosphatidylinositol-3-kinase (PI3KCIII) complex).^4^ In response to complicated extracellular signals, BECN1 is assembled into multiprotein complexes that includes ATG14L, UV radiation resistance-associated gene protein (UVRAG), AMBRA1, HMGB1, BCL-2 family proteins or prion protein (PRNP).^5, 6^

The caveolin family of integral membrane proteins have been identified as the major defining markers of caveolae. Caveolin proteins function as scaffolding proteins of specific lipids and play crucial roles in several signaling pathways.^7^ Among the three mammalian caveolin proteins (CAV1, CAV2 and CAV3), CAV1 is the principal component of caveolae membranes.^8^ CAV1 is involved in diverse signaling pathways with numerous interacting partners, including the EGF receptor and endothelial nitric oxide synthase.^9, 10^ Interestingly, CAV1 is phosphorylated at tyrosine-14 (Tyr14)^11^ and its multiple functions are highly regulated by this phosphorylation. The phosphorylation of CAV1 is essential in membrane trafficking and proper regulation of oxidative stress,^12^ and is also required for the interaction of CAV1 with other proteins, which is driven by changing caveolae morphology.^13^ Recent studies provided the evidence that CAV1 might participate in regulating autophagy under several stress conditions.^14, 15^ In addition, CAV1 is involved in the pathogenesis of ischemic injuries. Depletion of CAV1 markedly increases cardiac ischemia and reperfusion injuries, and cerebral ischemic damage.^16, 17^ Importantly, the phosphorylation of CAV1 contributes to protection against ischemic injuries.^18^ However, the molecular mechanisms underlying this protection are obscure.

Stroke is the second leading cause of mortality in adults and a major cause of disability worldwide.^19^ Approximately 85% of strokes are ischemic. Substantial efforts have been made to understand the molecular mechanisms of ischemia-induced cerebral damage and to develop neuroprotective agents for use in the treatment of strokes.^20^ During the ischemic insult, reactive oxygen species (ROS) are generated and cause oxidative stress.^21^ In general, ROS at physiological levels play an important role in normal cellular signaling to induce adaptive responses. However, high levels of ROS produced by dysfunctional mitochondria in response to ischemic stress eventually lead to cell death by inducing oxidative damage to susceptible proteins and lipids.^22^ To tolerate severe oxidative stress, cells operate a quality control system to clear damaged proteins and organelles, and thus maintain the overall health of tissues.

In the current study, we identify a novel role of CAV1 in autophagy activation under oxidative stress and ischemic injury. We show that the phosphorylation of CAV1 is essential for its interaction with BECN1/VPS34 and the initiation of autophagy. CAV1 deficiency thus leads to increased damage during oxidative stress and following cerebral ischemic injury in mice.

## Results

### CAV1 deficiency impairs autophagy activation following H_2_O_2_ treatment

With the aim of identifying novel regulators of autophagy under various stress conditions, we employed a functional screening approach using a cell-based assay. Previously, we identified PRNP as an autophagy activator and a BECN1-interacting protein.^6^ Our previous study also identified that CAV1 might be a putative PRNP-interacting protein.^6^ To understand this function of CAV1 in autophagy, we first addressed whether the ablation of CAV1 expression affected autophagy under various stress conditions. In these experiments, we utilized CRISPR/Cas9 technology to introduce a mutation (CAV1-KO) that abolished CAV1 expression in HeLa cells, a frequently utilized human cell line to study autophagy and mitophagy (Supplementary Figure S1a). Treatment with many autophagy-activating chemicals, such as Dulbecco’s modified Eagles medium (DMEM), Earle‘s Balanced Salt Solution, H_2_O_2_, CCCP and tunicamycin, increased the numbers of LC3 foci (APs) in HeLa cells. Of these, only LC3 foci formation in response to H_2_O_2_ treatment was abolished in our CAV1-KO cell line (Supplementary Figures S1c and d).

**Figure 1:**
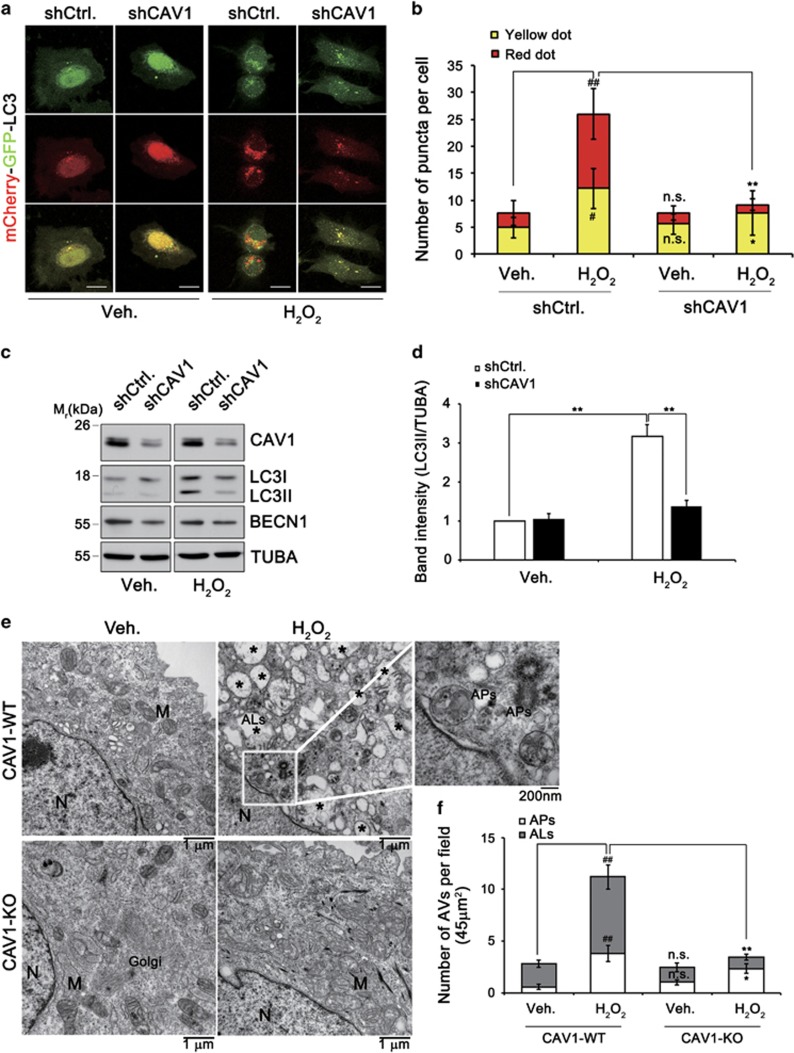
CAV1 knockdown impedes autophagy activation by H_2_O_2_. (**a** and **b**) Control (shCtrl.) and CAV1 knockdown (shCAV1) HeLa stable cells were transfected with mCherry-GFP-LC3 for 24 h and then treated with 3 mM H_2_O_2_ for 30 min. Cells were fixed with 4% paraformaldehyde for 5 min and then observed under a confocal microscope (scale bars=10 *μ*m) (**a**). The number of yellow (GFP and mCherry-double positive) or red puncta per cell in confocal images were quantified (mean values±S.D., *n*=10; ^#^*P*<0.05, ^##^*P*<0.005 *versus* veh. shCtrl.; *P<0.05, **P<0.005 *versus* H_2_O_2_ shCtrl.; NS, not significant *versus* veh. shCtrl.) (**b**). (**c** and **d**) Control (shCtrl.) and CAV1 knockdown (shCAV1) HeLa stable cells were treated with 3 mM H_2_O_2_ for 30 min and analyzed by western blotting (**c**). The signals on the blot were quantified using Science Lab software (Hercules, CA, USA) and the relative ratios of LC3-II to TUBA were calculated (mean values±S.D., *n*=3; ^**^P<0.005 *versus* shCtrl. cells) (**d**). (**e**) CAV1-WT and CAV1-KO HeLa cells were treated with 3 mM H_2_O_2_ for 30 min, and subjected to electron microscopic analysis. Autolysosomes (ALs, asterisks), mitochondria (M), autophagosomes (APs), golgi apparatus (Golgi) and nuclei (N) are indicated. (**f**) The numbers of cytoplasmic ALs and APs were counted (mean values±S.D., *n*=10; ^#^*P*<0.05, ^##^*P*<0.005 *versus* veh. CAV1-WT cells; *P<0.05, **P<0.005 *versus* H_2_O_2_-treated CAV1-WT cells; NS, not significant *versus* veh. CAV1-WT cells)

To evaluate the effect of H_2_O_2_ on autophagic flux in detail, we employed a mCherry-GFP-LC3 assay in which the appearance of yellow (mCherry and GFP) and red (mCherry) dots indicates the presence of LC3 in autophagosomes and autolysosomes (ALs), respectively.^23^ Treatment with H_2_O_2_ increased the numbers of both yellow and red dots in our control cells. On the contrary, this increase was not observed in CAV1 knockdown HeLa cells (Figures 1a and b). Western blot analysis showed that treatment with H_2_O_2_ significantly increased the level of LC3-II in control cells but not that in CAV knockdown HeLa cells (Figures 1c and d). We also found similar results in CAV1-KO primary cortical neurons (Supplementary Figure S1b). In addition, examination of intracellular structures using transmission electron microscopy (TEM) showed that treatment with H_2_O_2_ drastically increased the number of autophagic vacuoles surrounded by double-membrane structures (APs) and single membrane structures (ALs) in control HeLa cells (Figures 1e and f). On the contrary, these increases were not observed in CAV1-KO HeLa cells. These findings indicate that treatment with H_2_O_2_ increases autophagic flux and this effect is dependent upon CAV1 in HeLa cells.

### BECN1 and CAV1 interact together and colocalize in subcellular compartments under oxidative stress

**Figure 2:**
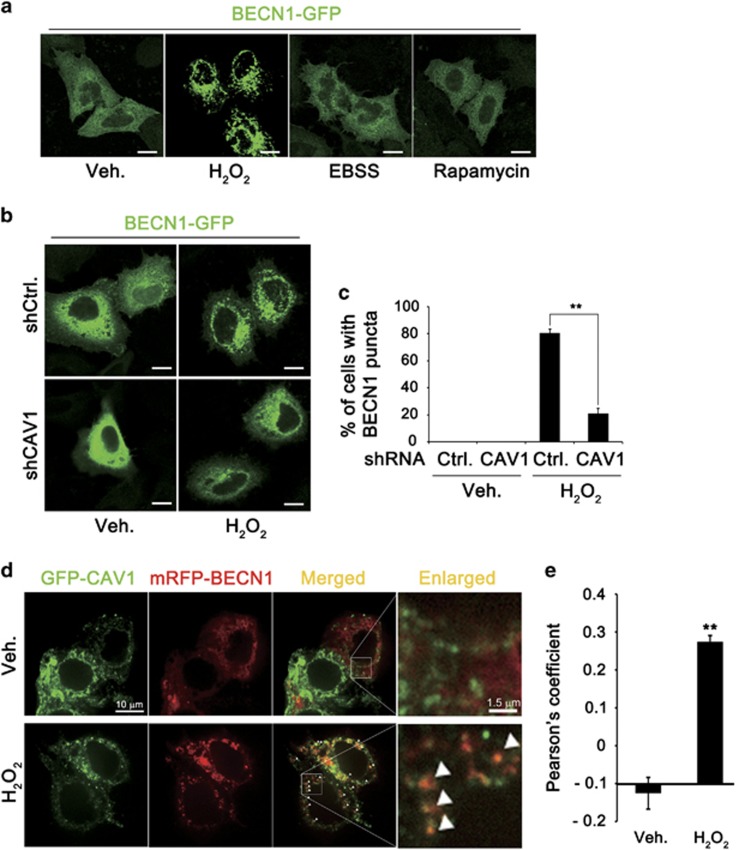
BECN1 translocates to CAV1-located subcellular compartment in response to H_2_O_2_. (**a**) HeLa cells were transfected with BECN1-GFP for 24 h, and then treated with 3 mM H_2_O_2_ for 30 min, EBSS for 2 h and 100 nM rapamycin for 1 h. Cells were fixed with 4% paraformaldehyde for 5 min and observed under a confocal microscope. (**b** and **c**) Control (shCtrl.) and CAV1 knockdown (shCAV1) HeLa stable cells were transfected with BECN1-GFP for 24 h. Cells were treated with 3 mM H_2_O_2_ for 30 min and observed under a confocal microscope (**b**). The number of cells with BECN1 puncta in confocal images, including (**b**), was quantified (mean values±S.D., *n*=10; **P<0.005 *versus* control cells) (**c**). (**d**) HeLa cells were transfected with both CAV1-GFP and BECN1-mRFP for 24 h and then treated with 1 mM H_2_O_2_ for 30 min. Cells were then observed under a confocal microscope (scale bars=10 *μ*m). (**e**) The Pearson’s coefficient between CAV1-GFP and BECN1-mRFP determined from confocal images, including (**d**), was calculated (mean values±S.D., *n*=5; **P<0.005 *versus* control cells)

To address whether CAV1 is required for subcellular distribution of BECN1 to regulate autophagic process, we examined the effect of H_2_O_2_ on the subcellular distributions of GFP-tagged autophagy proteins. The predicted subcellular localizations of ATG5-GFP, ATG9-GFP, ATG13-GFP and DFCP1-GFP in the cytosol, nucleus and ER, respectively, were not affected by treatment with H_2_O_2_ in HeLa cells (Supplementary Figure S2a). BECN1-GFP was detected with a diffuse distribution in the cytosol of untreated control cells and its localization was changed a little after treatment with Earle‘s Balanced Salt Solution or rapamycin. On the contrary, subcellular distribution of GFP-BECN1 was drastically changed to a dot-like pattern in the cytosol of HeLa cells after treatment with H_2_O_2_ (Figure 2a). Interestingly, silencing CAV1 expression in HeLa cells abolished the H_2_O_2_-induced change in BECN1 localization (Figures 2b and c). Further, fluorescence microscopy revealed that GFP-CAV1 signal overlapped with mRFP-BECN1 signal, but not with ATG5-GFP, ATG9-GFP and ATG13-GFP signals, following treatment with H_2_O_2_, whereas it appeared to partially colocalize with the early AP marker DFCP1 (Figures 2d, e and Supplementary Figure S2b). These results indicate that CAV1 is required for the change in BECN1 localization that follows oxidative stress.

**Figure 3:**
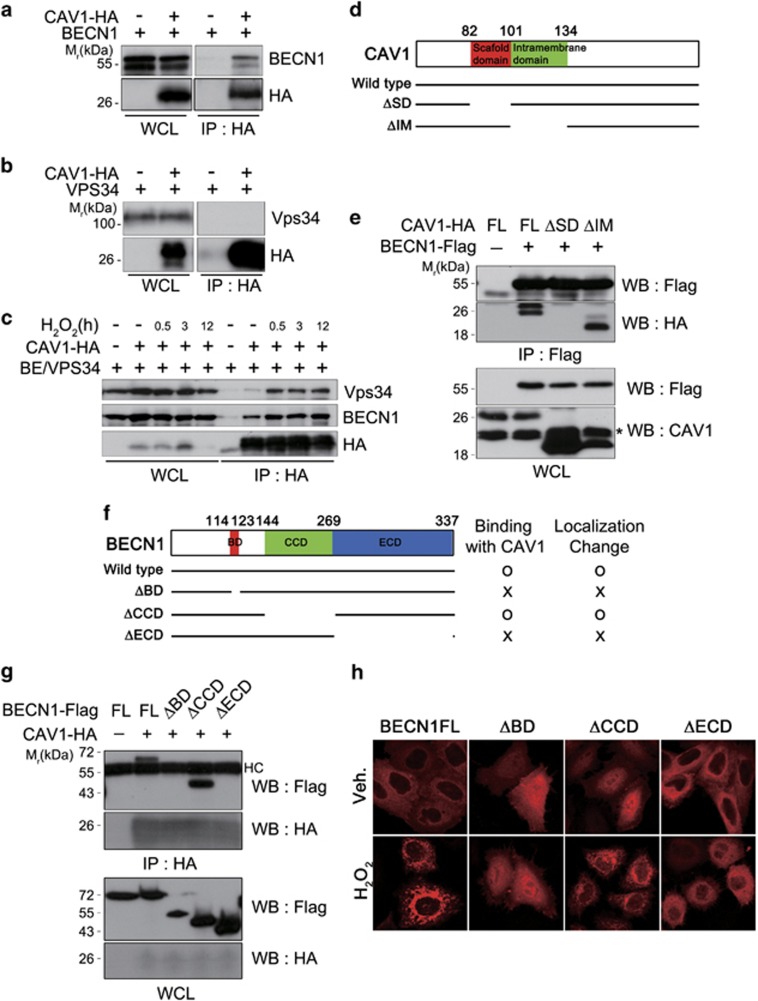
CAV1 binds to BECN1 for their colocalization in response to H_2_O_2_. (**a** and **b**) HEK293T cells were transfected with CAV1-HA/BECN1 (**a**) or CAV1-HA/VPS34 (**b**) for 24 h, and subjected to immunoprecipitation (IP) assay using anti-HA antibody. The immunoprecipitates and whole cell lysates (WCL) were analyzed with western blotting. (**c**) HEK293T cells were transfected with CAV1-HA, VPS34-flag and BECN1-flag for 24 h, and treated with 1 mM H_2_O_2_ for the indicated time. Cell lysates were subjected to IP assay using anti-HA antibody. (**d**) The diagram showing domain structure of CAV1 and its deletion mutants. (**e**) HEK293T cells were transfected with CAV1-HA (FL), CAV1ΔSD-HA (ΔSD) or CAV1ΔIM-HA (ΔIM) together with BECN1-flag as indicated. After 24 h, cell lysates were subjected to IP assay using anti-Flag antibody. Asterisk indicates endogenous CAV1. (**f**) Schematic representation of BECN1 and its deletion mutants and summary for its interaction with CAV1. (**g**) HEK293T cells were transfected with BECN1-Flag (FL), BECN1ΔBD-Flag (ΔBD), BECN1ΔCCD-Flag (ΔCCD) or BECN1ΔECD-Flag (ΔECD) with CAV1-HA for 24 h. Cell lysates were subjected to IP assay using HA antibody. (**h**) HeLa cells were transfected with BECN1-mRFP, BECN1ΔBD-mRFP (ΔBD), BECN1ΔCCD-mRFP (ΔCCD) or BECN1ΔECD-mRFP (ΔECD) for 24 h, and treated with 3 mM H_2_O_2_ for 30 min. Cells were then observed under a fluorescent microscope

The observation that BECN1 colocalized with CAV1 under oxidative stress led us to ask whether BECN1 interacts with CAV1. HEK293T cells were utilized to address this question because of high transfection efficiency. The results of co-immunoprecipitation (co-IP) assays showed that HA-tagged CAV1 could pull down BECN1 in HEK293T cells (Figure 3a). However, CAV1-HA did not robustly pull down VPS34, a component of the PI3KCIII complex, under basal condition (Figure 3b). We next examined whether CAV1-BECN1 binding is affected by H_2_O_2_, and found that the binding between CAV1 and BECN1 was enhanced in HEK293T cells within 30 min of treatment with H_2_O_2_. In this set, the interaction between CAV1-HA and VPS34 was very weak in untreated control cells, but was enhanced in cells treated with H_2_O_2_ (Figure 3c). These observations indicate that CAV1 forms a protein complex with BECN1 and VPS34 in response to oxidative stress. Moreover, colocalization of BECN1-mRFP with Atg14L-GFP, but not UVRAG-GFP, was increased following treatment with hydrogen peroxide (Supplementary Figures S3c and d). We thus addressed whether CAV1 forms a protein complex with other BECN1-interacting proteins, such as ATG14 and UVRAG. The results from IP assay revealed that CAV1-HA also interacts with ATG14-flag, but not with UVRAG-flag (Supplementary Figure S3a and b).

Next, we identified the binding domains of CAV1 and BECN1 that were important for their interaction. CAV1 contains two distinct domains, a scaffolding domain (SD, amino acids (aa) 82–101) and intramembrane (IM) domain (aa 101–134) (Figure 3d). Most CAV1-interacting proteins have canonical CAV1-binding motifs (CBMs) and interact with the SD of CAV1 through their CBMs.^24^ BECN1 is predicted to contain a BCL-2-binding domain (BD, aa 14–123), a coiled-coil domain (CCD, aa 144–269) and an evolutionarily conserved domain (ECD, aa 244–337) (Figure 3f).^25^ Co-IP assays using truncated protein constructs revealed that CAV1ΔIM bound to BECN1-Flag as much as full length CAV1, but CAV1ΔSD did not (Figure 3e). On the other hand, neither BECN1ΔECD nor BECN1ΔBD effectively bound to CAV1 (Figure 3g). These results indicate that the BD and ECD of BECN1 and the SD of CAV are required for their interaction. Further, fluorescence microscopy revealed a correlation between the subcellular localization patterns of BECN1 mutants and their ability to interact with CAV1 by co-IP. Like full length BECN1, the subcellular localization of CAV1-binding BECN1ΔCCD was affected by H_2_O_2_ treatment, whereas the localizations of the CAV1-binding defective BECN1ΔBD and BECN1ΔECD were not changed following H_2_O_2_ treatment (Figure 3h). Thus, we conclude that the binding of CAV1 to BECN1 is critical for the change in BECN1 localization in HeLa cells in response to oxidative stress.

### Mitochondrial localization of BECN1 under oxidative stress is CAV1-dependent

**Figure 4:**
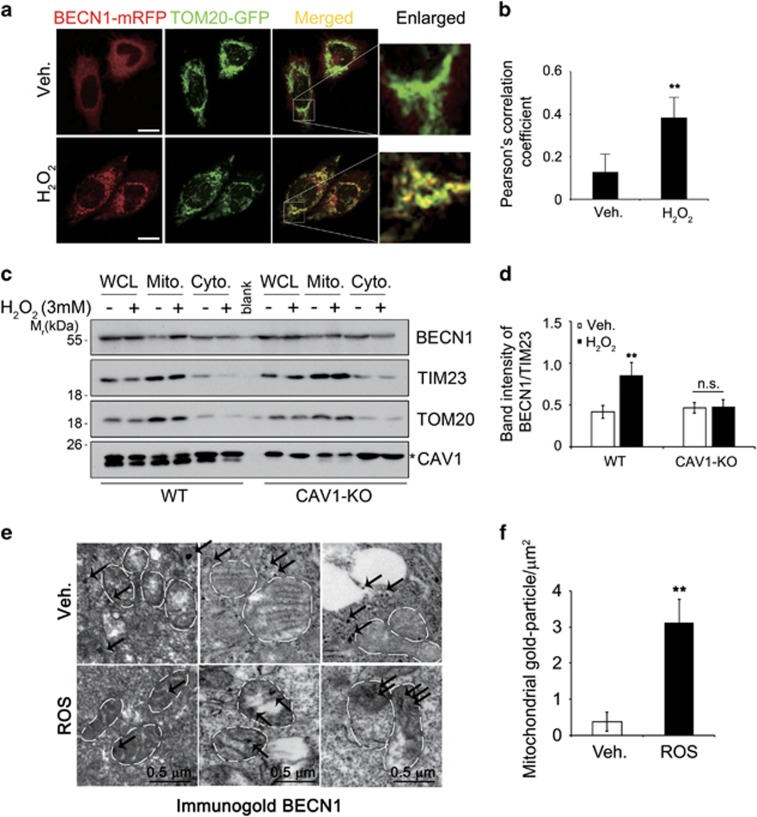
BECN1 translocates onto the mitochondria following H_2_O_2_ treatment. (**a**) HeLa cells were transfected with BECN1-mRFP and TOM20-GFP for 24 h, treated with 3 mM H_2_O_2_ for 30 min and observed under a confocal microscope (scale bars=10 *μ*m). (**b**) Mean values of Pearson’s correlation coefficient for colocalization between BECN1-mRFP and TOM20-GFP were calculated (*n*=5; **P<0.005 *versus* control cells). (**c**) Wild-type (WT) and CRISPR/Cas9 CAV1 (CAV1-KO) HeLa stable cells were treated with 3 mM H_2_O_2_ for 30 min and subjected to subcellular fractionation assay. The fractions were analyzed by western blotting. (**d**) The signals of BECN1 on the mitochondrial fraction (relative to TIM23) were quantified (mean values±S.D., *n*=3; **P<0.05 *versus* control cells). (**e**) HeLa cells were transfected with BECN1-flag for 24 h and treated with 5 mM H_2_O_2_ for 30 min. The localization of BECN1 was examined by immunogold electron microscopy using anti-Flag antibody. White broken circles show mitochondria and black arrows indicate BECN1-positive immunogold particle (scale bars=0.5 *μ*m). (**f**) The numbers of gold particles on the mitochondria per *μ*m^2^ were counted (mean values±S.D., *n*=8; **P<0.05 *versus* control cells)

We next sought to identify the subcellular localization of BECN1 under oxidative stress. Colocalization assays utilizing GFP-tagged organelle-specific proteins revealed that under oxidative stress, BECN1-mRFP most strongly overlapped with GFP-tagged TOM20, a mitochondrial protein (Figures 4a and b). Conversely, BECN1 did not much colocalize with the other organelle-marker proteins we tested, including an ER-retention signal-tagged RFP and an early endosome marker (RAB5-GFP) (Supplementary Figures S4a and b).^26^ In addition, the results of a subcellular fractionation assay were consistent to a mitochondrial localization of BECN1. Notably, H_2_O_2_ treatment significantly increased the level of BECN1 in the mitochondria-rich fractions of HeLa cell extracts, but not in the fractions prepared from CAV1-KO HeLa cells (Figures 4c and d). As previously reported,^27^ CAV1 was also detected in the mitochondria-rich fractions, but its levels in these fractions were not affected by H_2_O_2_ treatment. Finally, TEM revealed that immunogold-labeled BECN1 was detected in the cytosol of HeLa cells, but became detectable within the mitochondria under oxidative stress (Figures 4e and f). In sum, our results show that BECN1 translocates to the mitochondria in a CAV1-dependent manner under oxidative stress.

Next, we assessed a possible role for mitochondrial BECN1 in autophagy under oxidative stress. We evaluated its function in the selective autophagic degradation of mitochondria (mitophagy) using well-established assays.^28^ Confocal microscopy showed that treatment of HeLa cells with H_2_O_2_ increased the colocalization of GFP-LC3 with mito-RFP. This colocalization was markedly reduced in CAV1-depleted HeLa cells (Supplementary Figure S5). However, examination of mitochondrial protein levels by western blot revealed that the mitochondrial inner-membrane protein TIM23 was degraded following H_2_O_2_ treatment, and this degradation occurred with similar kinetics in control and CAV1-depleted HeLa cells (Supplementary Figure S6). Together, these data show that mitochondrial BECN1 is likely to function in GFP-LC3 recruitment to mitochondria during autophagy, but not during mitophagy.

### CAV1 phosphorylation is required for its binding to BECN1 and autophagy initiation

**Figure 5:**
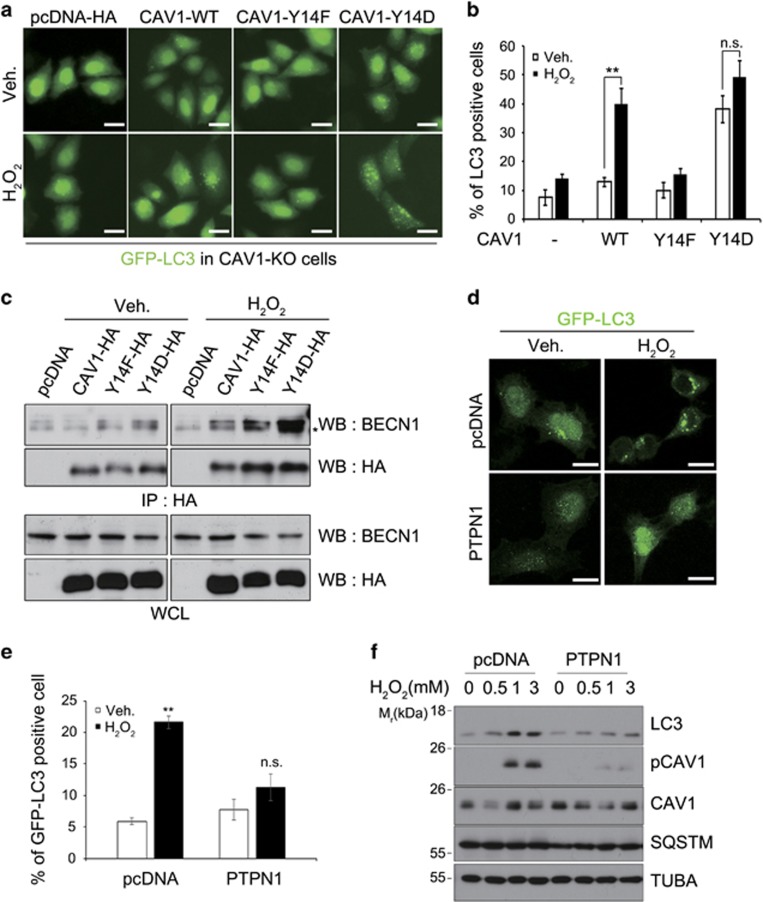
Phosphorylated CAV1 interacts with BECN1 and activates autophagy under oxidative stress. (**a** and **b**) CRISPR/Cas9 CAV1 (CAV1-KO) HeLa cells were transfected with GFP-LC3 and pcDNA, CAV1-HA, CAV1-Y14F-HA or CAV1-Y14D-HA for 24 h, and treated with 3 mM H_2_O_2_ for 1 h. Cells were observed under a fluorescent microscope (**a**). The ratios of LC3 dot-positive cells on the microscopy images, including (**a**), were determined (mean values±S.D., *n*=3; **P<0.005 *versus* control cells) (scale bars=10 *μ*m) (**b**). (**c**) HEK293T cells were transfected with pcDNA, CAV1-HA, CAVY14F-HA and CAV1-Y14D-HA for 24 h, and then treated with 1 mM H_2_O_2_ for 30 min. Cell lysates were analyzed by IP assay using anti-HA antibody. (**d** and **e**) HeLa cells were transfected with GFP-LC3, and either pcDNA-HA or PTPN1 for 24 h, and then treated with 3 mM H_2_O_2_ for 30 min. Cells were observed under a confocal microscope (scale bars=10 *μ*m) (**d**). The ratios of LC3 dot-positive cells on the microscopy images were determined (mean values±S.D., *n*=10; **P<0.005 *versus* control cells) (**e**). (**f**) HeLa cells were transfected with pcDNA or PTPN1 for 24 h, and then treated with the indicated doses of H_2_O_2_ for 30 min. Cell lysates were examined by western blot analysis. NS, not significant

Because the levels of CAV1 at the mitochondria were not changed by oxidative stress, we hypothesized that CAV1 might be modified to recruit BECN1 in response to this stress. As reported,^12^ CAV1 was phosphorylated at Tyr14 in HeLa cells following treatment with 1 mM H_2_O_2_ (Supplementary Figures S7a and b). We thus investigated whether the phosphorylation of CAV1 was required for autophagy regulation. In CAV1-KO cells, we found that expression of a CAV1 mutant mimicking constitutive phosphorylation (CAV1-Y14D) increased GFP-LC3 puncta formation, whereas a CAV1 mutant that could not be phosphorylated (CAV1-Y14F) failed to increase GFP-LC3 puncta formation (Figures 5a and b). In addition, co-IP assays revealed that CAV1-Y14D interacted with BECN1 and this interaction was drastically increased by oxidative stress (Figure 5c). On the other hand, there was no such increase in the interaction between CAV1-Y14F and BECN1. As previously reported,^7^ CAV1 was phosphorylated at Tyr14 by overexpression of wild-type SRC kinase (WT-SRC) and more drastically by CA-SRC, a constitutively active form of SRC (Supplementary Figure S7d). In this assay, we also found that LC3-II conversion was increased by WT-SRC and more by CA-SRC. Further, ectopic expression of Tyr-protein phosphatase non-receptor type 1 (PTPN1), a phosphatase acting on CAV1 (Figure 5f),^29^ inhibited GFP-LC3 puncta formation and LC3-II conversion under oxidative stress (Figures 5d–f). Together, these results indicate that the phosphorylation of CAV1 at Tyr14 is required for the interaction of CAV1 with BECN1 to activate autophagy.

We next investigated whether the phosphorylation of CAV1 is required for the recruitment of BECN1 to mitochondria under oxidative stress. Unlike CAV1, ectopically expressed CAV1-Y14D slightly affected the localization of BECN1-mRFP to the mitochondria in untreated control cells (Supplementary Figure S7c). Interestingly, treatment with H_2_O_2_ more drastically increased the colocalization of CAV1-Y14D with BECN1-mRFP than that of CAV1 with BECN1-mRFP at the mitochondria. On the other hand, ectopic expression of CAV1-Y14F inhibited the translocation of BECN1-mRFP to the mitochondria even under oxidative stress (Supplementary Figure S7c). Furthermore, unlike CAV1 that was found in all fractions, including cytosols, light membrane and mitochondria-rich fraction, the phosphorylated CAV1 was detected only in the mitochondria-rich fraction under oxidative stress (Supplementary Figure S7c). These findings illustrate the importance of CAV1 phosphorylation at Tyr14 in mediating the mitochondrial localization of BECN1 during oxidative stress.

### Loss of CAV1 impairs autophagy and increases infarct area following cerebral ischemia

Brain ischemia initiates a complex cascade of metabolic events, several of which generate free radicals to cause oxidative stress and damage.^30^ We therefore investigated the role of CAV1 in autophagy activation and stroke *in vivo* using a mouse model. We first examined the phosphorylation of CAV1 under conditions of hypoxia and found that CAV1 was phosphorylated at Tyr14 (Supplementary Figure S8a). Simultaneously, LC3-II conversion was increased in hypoxic HeLa cells. As reported elsewhere,^18^ we found the phosphorylated CAV1 in the region of the mouse brain ipsilateral to the cerebral ischemia after middle cerebral artery occlusion (MCAO) (Supplementary Figures S8b and c).^31^ As seen in H_2_O_2_ treatment, LC3-II conversion was enhanced in the damaged region compared to contralateral region of the mouse brain. The results show that CAV1 is phosphorylated, and autophagy is activated during hypoxia and ischemia.

**Figure 6:**
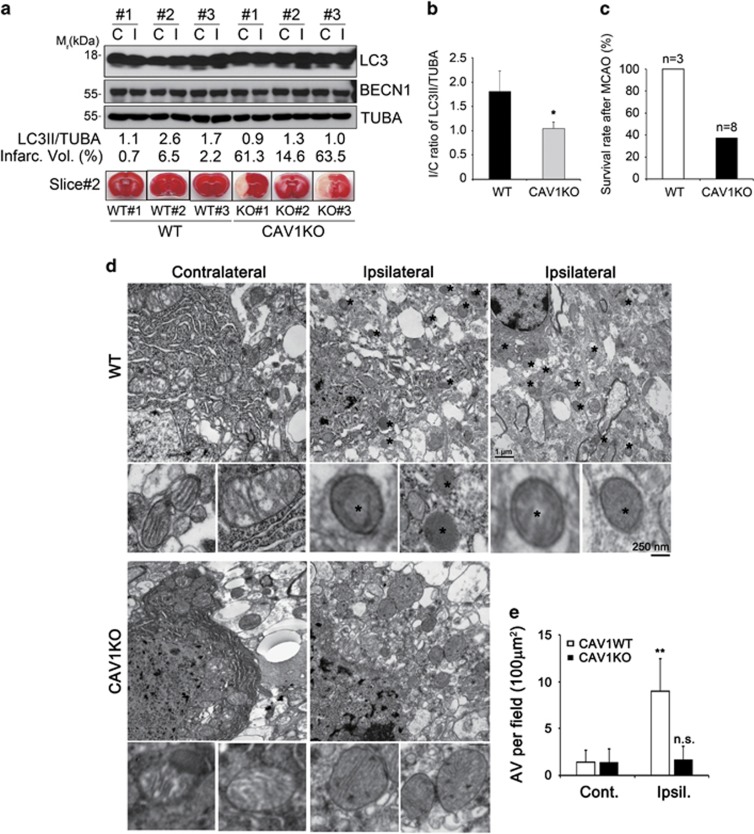
CAV1 knockout markedly limits autophagy activation in mouse cerebral ischemia model. (**a**–**e**) CAV1-WT and CAV1-KO mice were subjected to MCAO for 30 min and reperfusion for 24 h. Contralateral (C) and ipsilateral (I) tissues of the mouse brain were coronally sectioned and stained with 2 % TTC. The infarct volume was determined by measuring infarct size relative to normal in the slice (**a**, lower). Same tissue extracts were examined with western blot analysis using the indicated antibodies. The LC3-II signals on the blot were quantified using Science Lab software and the relative ratios of LC3-II to TUBA are shown at the bottom of the blot (**a**, upper). The averages of the three relative ratios were calculated (mean values±S.D., *n*=3; *P<0.05 *versus* control cells) (**b**). The survival rates of CAV1-WT (*n*=3) and KO (*n*=8) mice after MCAO surgery were calculated (**c**). Ultrastructural features of infarct area in the CAV1-WT and CAV1-KO brain were analyzed by electron microscopic analysis. Asterisks indicate autophagic vacuoles (AVs) and mitochondria are also shown in enlarged view (**d**). The numbers of AVs per fixed field (the area=100 *μ*m^2^) were calculated (mean values±S.D., *n*=15; **P<0.005; NS, not significant *versus* contralateral) (**e**)

We hypothesized that CAV1 deficiency might influence cell death during oxidative stress and ischemic damage. To address this point, we performed cell death assay after treatment with H_2_O_2_. The results revealed that treatment with H_2_O_2_ for 1 and 2 h induced 16 and 35% of cell death in CAV1-WT HeLa cells and 26 and 50% in CAV1-KO HeLa cells, respectively, indicating that the depletion of CVA1 functions to exacerbate death of HeLa cells (Supplementary Figure S10). Moreover, to evaluate the role of CAV1 in autophagy activation and ischemic damage, we employed an MCAO assay in *Cav1* knockout mice (*Cav1*^−/−^ mice). At 24 h after MCAO, the measured infarct areas were much larger in the brains of *Cav1*^−/−^ mice than in the brains of WT mice (Figure 6a,Supplementary Figures S9a and b), consistent to the previous report.^16^ Accordingly, the viability of *Cav1*^−/−^ mice was reduced to 40% after MCAO, while WT mice were all survived (Figure 6c). In addition, examination of LC3-II conversion showed that the ratio of LC3-II to *α*-tubulin ratio increased by 40% in the ipsilateral region of WT, but not in *Cav1*^−/−^ mice (Figure 6b). Further, from ultrastructural analysis of penumbra area by electron microscopy, many vacuolar structures that are reminiscent of AVs were detected in WT mice (Figures 6d, e and Supplementary Figure S11). On the contrary, such vacuolar structures were not observed in the penumbra area of *Cav1*^−/−^ mouse brains. These results suggest that CAV1 is essential for mediating autophagy under cerebral ischemic stress and for protecting the brain against ischemic damage.

## Discussion

Oxidative stress has been reported as an inducer of autophagy, and autophagy in turn contributes to clearing irreversibly oxidized biomolecules.^32^ While the precise molecular mechanisms by which oxidative stress activates autophagy are still unclear, we report that CAV1 positively regulates autophagy through interacting with BECN1 under oxidative stress and following cerebral ischemic injury in mice. CAV1 recruits many interacting partners with aromatic amino acid-rich CBMs, and with these partners CAV1 regulates various cellular signaling pathways, including those activated in response to oxidative stress.^33^ While there is no known CBM in the BD of BECN1, the ECD of BECN1 has an aromatic amino acid-rich motif (aa 354–363) similar to the putative CAV1-binding sequence (фXфXXXXф, ф is an aromatic and X an unspecified amino acid).^34, 35^ BECN1 regulates autophagy through interactions with its binding partners under various stresses or in response to various signals.

It was previously reported that CAV1 negatively regulates autophagy under basal or nutrient-deprivation conditions through interacting with ATG12–ATG5 or modulating lysosomal function.^14, 36^ In contrast, CAV1 was also shown to positively regulate 17*β*-estradiol-mediated autophagy and autophagy-mediated claudin-5 degradation.^15, 37^ Therefore, the question of how CAV1 can regulate autophagy negatively or positively might depend on the context that arises. Our observation that the phosphorylation of CAV1 is critical for autophagy regulation might be an important clue that should help future studies dissect the distinct functions of CAV1 in autophagy. Further, our finding that CAV1 interacts with BECN1 in a phosphorylation-dependent manner is a key insight into the mechanism of CAV1 activity. We show here that CAV1-binding to BECN1 involves the BD and ECD of BECN1, and requires the SD of CAV1. Future studies should address exactly how phosphorylation of the N-terminal region of CAV1, on a residue outside the SD, affects its binding with BECN1.

Interestingly, we found that both BECN1 and GFP-LC3 were translocated to the mitochondria under oxidative stress in a CAV1-dependent manner. Since many studies have used colocalization of GFP-LC3 with mitochondrial marker proteins as an indicator of mitophagy,^28^ we also initially believed that CAV1-dependent mitochondrial localization of BECN1 might play a role in mitophagy. On the basis of mitochondria degradation assay in CAV1-KO cells, however, we concluded that CAV1 does not affect mitophagy under oxidative stress. Therefore, why BECN1 is recruited into the mitochondria under oxidative stress remains an important question. The mitochondria are highly prone to damage compared to other organelles and especially prone to damage by oxidative stress.^38^ This damage might provide a platform to generate a signal for the initiation of complicated types of autophagy or the assembly of APs. Oxidative stress might induce LC3-independent alternative autophagy^39^ as well as conventional autophagy. Thus, mitochondrial degradation can still occur in CAV1-KO HeLa cells under oxidative stress, though the colocalization between mitochondria and LC3 is drastically decreased in this condition. This point of view is interesting but needs to be further addressed in future. In addition, like the early AP marker DFCP1, the autophagy proteins ATG9 and cleaved-ATG5 have previously been shown to localize onto the mitochondria during autophagy.^40, 41^ Consistent to our proposal, other reports have shown that mitochondria and/or mitochondria-associated membranes participate in the formation of APs.^42, 43^ While many details remain to be addressed, we believe that CAV1 acts as an anchor protein to recruit BECN1 and ATG proteins to the mitochondria to initiate or stimulate autophagy under oxidative stress.

Our results show that CAV1 has a protective role in ischemic injury. The cerebral volume of infarction was drastically increased in *Cav1*^−/−^ mice, as compared with that of WT or *Cav2*^−/−^ mice.^16^ The detection of LC3-II in the brain tissue by western blot is challenging due to the tremendous expression of LC3-I. Despite this, we detected a clear increase in LC3-II levels in the ipsilateral ischemic hemisphere of WT mouse brains, but not in *Cav1*^−/−^ mouse brains. This result supports the model that autophagy activation by CAV1 protects tissue from ischemic damage. The levels of CAV1 as a regulator of BECN1 appear to be differentially modulated in the ischemic core and penumbra areas of the ischemic brain.^44^ Additional autophagy assays using TEM to detect AVs in penumbra area show drastic differences in the number of AVs between WT and *Cav1*^−/−^ mice under cerebral ischemic injury. We conclude that autophagy activation by CAV1 under cerebral ischemic injury protects against ischemic damage.

**Figure 7:**
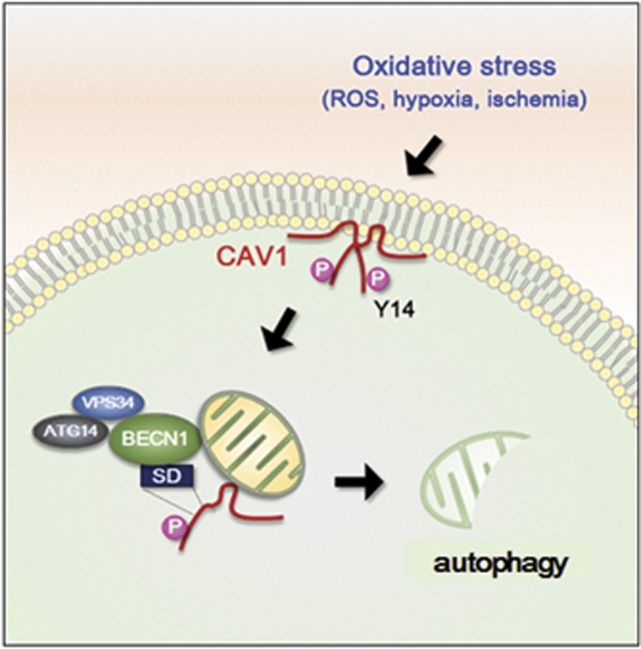
A proposed role of phosphorylated CAV1 in autophagy activation. Oxidative stresses, such as hydrogen peroxide, hypoxia and ischemic injury, induce the phosphorylation of CAV1 at 14-Tyr. Phosphorylated CAV1 then interacts with BECN1/VPS34 complex and recruits this complex onto the mitochondria to activate autophagy

In summary, we demonstrate that CAV1 acts as a sensor that detects oxidative stress or ischemic injury, and subsequently interacts with BECN1 to initiate autophagy and protect relevant cells from damage (Figure 7). In this process, the phosphorylation of CAV1 is crucial for activation of BECN1-mediated autophagy.

## Materials and methods

### Reagents and plasmid constructions

The following chemicals used include rapamycin, tunicamycin (Sigma-Aldrich, St. Louis, MO, USA), carbonyl cyanide m-chlorophenyl hydrazone (CCCP; Calbiochem, San Diego, CA, USA) and hydrogen peroxide (H_2_O_2_, Sigma-Aldrich). Caveolin1 deletion mutants were generated by subcloning the PCR products (ΔSD (SD (aa 82–101) deletion)/ΔIM (IM domain (aa 101–134) deletion)) into pcDNA3-HA and pEGFP. Beclin1 deletion mutants, pEGFP-LC3, Atg5-pEGFP and mCherry-GFP-LC3 have previously been described.^6^ Atg14L and UVRAG were subcloned into Flag-tagging vector. For Caveolin1 shRNA, target sequences were cloned into pSuper-Neo vector (pshRNA, OligoEngine, Seattle, WA, USA). The target sequences for Caveolin1 were (shCav1: 5′-GCATTTGGAAGGCCAGCTT-3′, 5′-AAGCTGGCCTTCCAAATGC-3′). For CRISPR/Cas9 Caveolin1, target sequences were cloned into lentiCRISPR v2 vector (Addgene, Cambridge, MA, USA). The target sequences for Caveolin1 were gCAV1: 5′-CACCGGTTTAGGGTCGCGGTTGACC-3′, 5′-AAACGGTCAACCGCGACCCTAAACC-3′.

### Antibodies, immunoprecipitation assay and western blot analysis

The primary antibodies used include anti-LC3B, anti-beclin1 (Novus Biologicals, Littleton, CO, USA), anti-caveolin1 (Abcam, Cambridge, UK), anti-TIMM23, anti-phospho-caveolin1 (BD Bioscience, San Jose, CA, USA), anti-HA, anti-Vps34 (Cell Signaling Technology, Danvers, MA, USA), anti-tubulin, anti-Flag (Sigma-Aldrich), anti-p62 (Abnova, Taiwan) and anti-TOMM20 (Santa Cruz Biotechnology, Dallas, TX, USA). Immunoprecipitation assays were performed as previously described;^6^ briefly, cells were lyzed with modified RIPA buffer (10 mM Tris-HCl, 150 mM NaCl, 5 mM EDTA, 1 mM Na_3_VO_4_, 1% CHAPS). After pull-down with the appropriate antibodies, same amounts of protein were separated by SDS–polyacrylamide gel electrophoresis (PAGE) and transferred onto the polyvinylidene fluoride membrane (Millipore, Billerica, MA, USA). Immunoblot analysis was then performed and visualized by the enhanced chemiluminescence method.

### Cell culture, transfection and generation of CAV1-KO stable cell lines

SH-SY5Y cells (human neuroblastoma cells), HEK293T cells (human embryonic kidney cells) and HeLa (human cervical carcinoma) cells were cultured in DMEM (Hyclone, Logan, UT, USA) with 10% (v/v) fetal bovine serum (Hyclone). To generate CAV1-KO HeLa cells stably expressing CRISPR/Cas9 CAV1, cells were transfected with CRISPR/Cas9 CAV1 (Polyfect, Qiagen, Hilden, Germany) for 24 h and incubated with 1 mg/ml puromycin (Invitrogen, Carlsbad, CA, USA) for 2 more weeks to generate stable cells. The stable cells were then confirmed by western blotting following the standard protocol.

### Maintenance of *Cav1*-KO mice

We used *Cav1*-KO mice (Jax, stock number: 004585; https://www.jax.org/strain/004585). All mice were maintained under a 12 :12 h light/dark cycle. All of the experiments were performed on C57BL/6J mice and were approved by the Seoul National University Standing Committees on Animals.

### Subcellular fractionation assay

Fractionation of cell homogenates was carried out as previously described.^45^ In brief, both wild-type and CAV1 knockout HeLa cells were washed, collected and homogenized with a sonicator (Vibra-Cell, SONICS, Newtown, CT, USA) three times for 10 s in PBS containing proteases cocktail. Unbroken cells and nuclei were removed by centrifugation at 1000 g for 10 min. The post-nuclear supernatant fraction was pelleted by further centrifugation at 10 000 g for 15 min to obtain the mitochondria pellet. After washing the pellet twice with PBS, the resulting pellet (mitochondrial membrane) was resuspended in SDS sample buffer. The supernatant (cytosol) in the first centrifugation was added with concentrated SDS sample buffer.

### Hypoxia

Cells were exposed to hypoxia by incubating at 37 °C in 1% O_2_/5% CO_2_/94% N_2_ anaerobic condition in an air-tight modular incubator chamber (Billups-Rothenberg Inc., San Diego, CA, USA).^46^

### TCC staining

MCAO mouse brain slices were incubated in 2% 2,3,5-triphenyltetrazolium chloride (Sigma-Aldrich, St. Louis, MO, USA) in PBS for 20 min at room temperature.

### Transmission electron microscope analysis

Cells were fixed with 2% paraformaldehyde/2% glutaraldehyde in 0.1 M phosphate buffer (PB, pH 7.4), followed by 1% OsO_4_. Cells were further dehydrated with a graded series of ethanol and embedded in epoxy resin. Ultrathin sections were stained with uranyl acetate and lead citrate for observation under a JEM1010 transmission electron microscope (JEOL, Tokyo, Japan).

### Immunogold TEM

Immunogold TEM was carried out as previously described.^47^ In brief, HeLa cells were transfected with BECN1-flag for 24 h, and then fixed in 4% paraformaldehyde and 0.1% glutaraldehyde for 30 min. Cells were washed in PBS buffer three times, incubated in 0.1 M Na-PB containing 0.25% saponin and 5% BSA for 30 min, and then incubated in PB containing 0.005% saponin, 10% BSA and 0.1% cold water fish skin gelatin. Cells were treated with mouse monoclonal antibody against flag (Sigma-Aldrich) in the blocking solution overnight. Cells were then washed in PB containing 0.005% saponin for 5 min three times and incubated with anti-mouse IgG that was conjugated to colloidal gold (10 nm diameter) in the blocking solution for 2 h. Cells were washed with PB for 5 min three times and fixed with 1% glutaraldehyde in PB for 10 min. After washing in distilled water, cells were postfixed in 0.5% OsO_4_ for 2 h at 4 °C, washed in distilled water, incubated with 50% ethanol for 10 min and stained with 2% uranyl acetate in 70% ethanol for 2 h. Cells were further dehydrated with a graded series of ethanol and were embedded in epoxy resin. Ultrasections were doubly stained with uranyl acetate and lead citrate.

### MCAO

CAV1-WT or KO male mice (3- to 4-month old) were anesthetized with intraperitoneal injection of zoletil/rompun/PBS mixture and transient MCAO was conducted as previously described.^48^ Briefly, the right common carotid artery was exposed through a midline incision in the neck and unilateral MCAO was conducted by inserting a 6-0 silicone rubber-coated monofilament (Doccol Corp., Sharon, MA, USA). The MCAO was occluded for 30 min and then the suture was withdrawn to allow 24 h of reperfusion before killing.

### Statistics

All experiments were performed in triplicate parallel instances and repeated at least three times. Statistical analyses were carried out using the Microsoft Office 2013 Excel software package (Microsoft Corporation, Redmond, WA, USA). Mean values were compared using unpaired *t*-tests.
