# Supplementary material for: Phosphorylated CAV1 activates autophagy through an interaction with BECN1 under oxidative stress
Source: Cell Death Dis. 2017 May 25;8(5):e2822–. doi: 10.1038/cddis.2017.71 (PMC5520747; doi:10.1038/cddis.2017.71)
Supplement: Supplementary Figure legend [file cddis201771x1.docx]

**Supplementary figure legend**

**Supplementary Figure S1. CAV1 knockout selectively impedes autophagy activation by H_2_O_2_.** (a) Wild-type (WT) and CRISPR/Cas9 CAV1 (CAV1-KO) HeLa stable cells were treated with 1 mM H_2_O_2_ for 1 h and then subjected to western blot analysis. (b) WT and CAV1-KO primary cortical neurons were treated with 3 mM H_2_O_2_ for 30 min and analyzed by Western blotting. (c and d) WT and CAV1-KO HeLa stable cells were transfected with GFP-LC3. After 24 h, cells were left untreated (Veh.) or incubated with serum-free DMEM for 6 h, amino acid-free EBSS for 3 h, 1 mM H_2_O_2_, 2 μg/mL tunicamycin (Tuni.) or 100 μM CCCP for 2 h, and then observed under a fluorescent microscope (c). The number of cells with GFP-LC3 dots were quantified (mean values ± S.D., *n* = 3; ***p* < 0.005 versus control cells) (d).

**Supplementary Figure S2. Subcellular localization of autophagy-related proteins under oxidative stress.** (a) HeLa cells were transfected with ATG5-GFP, ATG9-GFP, ATG13-GFP and DFCP1-GFP for 24 h and treated with 3 mM H_2_O_2_ for 30 min. Cells were observed under a fluorescence microscope. (b) HeLa cells were transfected with CAV1-mRFP, ATG5-GFP, ATG9-GFP, ATG13-GFP and DFCP1-GFP for 24 h, as indicated, and treated with 3 mM H_2_O_2_ for 30 min. Cells were observed under a fluorescence microscope.

**Supplementary Figure S3. ATG14 interacts with CAV1.** (a and b) HEK293T cells were cotransfected for 24 h with pcDNA-HA, BECN1-HA, CAV1-HA and ATG14L-flag (a) or pcDNA-HA, BECN-HA, CAV1-HA and UVRAG-flag (b) as indicated manner. Cell lysates were then subjected to immunoprecipitation (IP) analysis using anti-HA antibody. The immunoprecipitates and whole cell lysates (WCL) were analyzed by western blotting. (c and d) HeLa cells were transfected for 24 h with BECN1-mRFP, Atg14L-GFP (c) and UVRAG-GFP (d) as indicated. Cells were treated with vehicle (Veh.) or 3 mM hydrogen peroxide for 30 min, and then observed under confocal microscope.

**Supplementary Figure S4. BECN1 does not colocalize with RAB5-GFP and ER-RFP proteins under oxidative stress.** (a and b) HeLa cells were transfected with BECN1-mRFP and RAB5-GFP (a) or BECN1-GFP and ER-RFP (b) for 24 h. Cells were untreated (Veh.) or treated with 3 mM hydrogen peroxide for 30 min and then observed under a confocal microscope.

**Supplementary Figure S5. CAV1 is required for the translocation of GFP-LC3 onto the mitochondria in response to H_2_O_2_.** Control (Ctrl.) and CRISPR/Cas9 CAV1 (gCAV1) HeLa stable cells were transfected with GFP-LC3 and mito-RFP for 24 h. Cells were treated with 3 mM hydrogen peroxide for 30 min and observed under a confocal microscope.

**Supplementary Figure S6. CAV1 knockout does not affect mitochondrial degradation by H_2_O_2_.** Wild-type (WT) and CRISPR/Cas9 CAV1 (CAV1-KO) HeLa stable cells were treated with H_2_O_2_ for the indicated doses and times and then subjected to western blot analysis.

**Supplementary Figure S7. Phosphorylated CAV1 colocalizes with BECN1 under oxidative stress.** (a) HeLa cells were treated with 0.3 or 1 mM H_2_O_2_ for 30 min or 10 μM CCCP for 3 h. Cell lysates were examined by Western blot analysis. (b) HeLa cells were treated for 30 min with the indicated concentrations of H_2_O_2_. Cell lysates were analyzed with western blotting. (c) HeLa cells were transfected with BECN1-mRFP and CAV1WT-GFP, CAV1Y14F-GFP or CAV1Y14D-GFP for 24 h, treated with 3 mM H_2_O_2_ for 30 min and observed under a fluorescence microscope. (d) HeLa cells were transfected with pcDNA, WT-SRC or CA-SRC for 24 h. Cell lysates were subjected to Western blot analysis. (e) HeLa cells were treated with 3 mM H_2_O_2_ for 30 min. Cell lysates were analyzed by subcellular fractionation assay. The fractions were then examined by Western blot analysis.

**Supplementary Figure S8. Both phosphorylated CAV1 and autophagy activation are detected under hypoxic and ischemic conditions.** (a) HeLa cells were treated with 3 mM H_2_O_2_ for 30 min or hypoxia (1 % oxygen) for 24 h and subjected to western blot analysis. (b and c) Mice were subjected to MCAO surgery. At 24 h after cerebral ischemic damage, coronal sections of mouse brain were stained with 2 % TTC (b) or tissue extracts from contralateral (c) and ipsilateral (I) regions were prepared and subject to western blot analysis (c).

**Supplementary Figure S9. CAV1 knockout drastically increases infarction area in mouse cerebral ischemia model.** (a) Wild-type (WT) and CAV1-KO mice were subjected to MCAO for 30 min and reperfusion for 24 h. Coronal sections of mouse brain were stained with 2 % TTC. (b) Infarction volume on the staining images was determined (mean values ± S.D., *n* = 3; *P* value is donated).

**Supplementary Figure S10. CAV1 deficiency increases cell death during oxidative stress.** (a and b) CAV1-WT and CAV1-KO HeLa cells were treated with 3 mM H_2_O_2_ for the indicated times, stained with propodium iodide (PI), and then observed under fluorescence microscope (a). The PI-positive cells were counted (mean values ± S.D., n = 3; *p < 0.05, **p < 0.005 versus CAV1-WT).

**Supplementary Figure S11. CAV1 knockout attenuates autophagy activation in mouse cerebral ischemia model.** CAV1-WT and CAV1-KO mice were subjected to MCAO for 30 min and reperfusion for 24 h. Ultrastructural features of infarct area in CAV1-WT and CAV1-KO brain were observed by electron microscopic analysis.
