# Supplementary material for: Phosphorylated CAV1 activates autophagy through an interaction with BECN1 under oxidative stress
Source: Cell Death Dis. 2017 May 25;8(5):e2822–. doi: 10.1038/cddis.2017.71 (PMC5520747; doi:10.1038/cddis.2017.71)

Supplementary Figure 1. Nah et al.

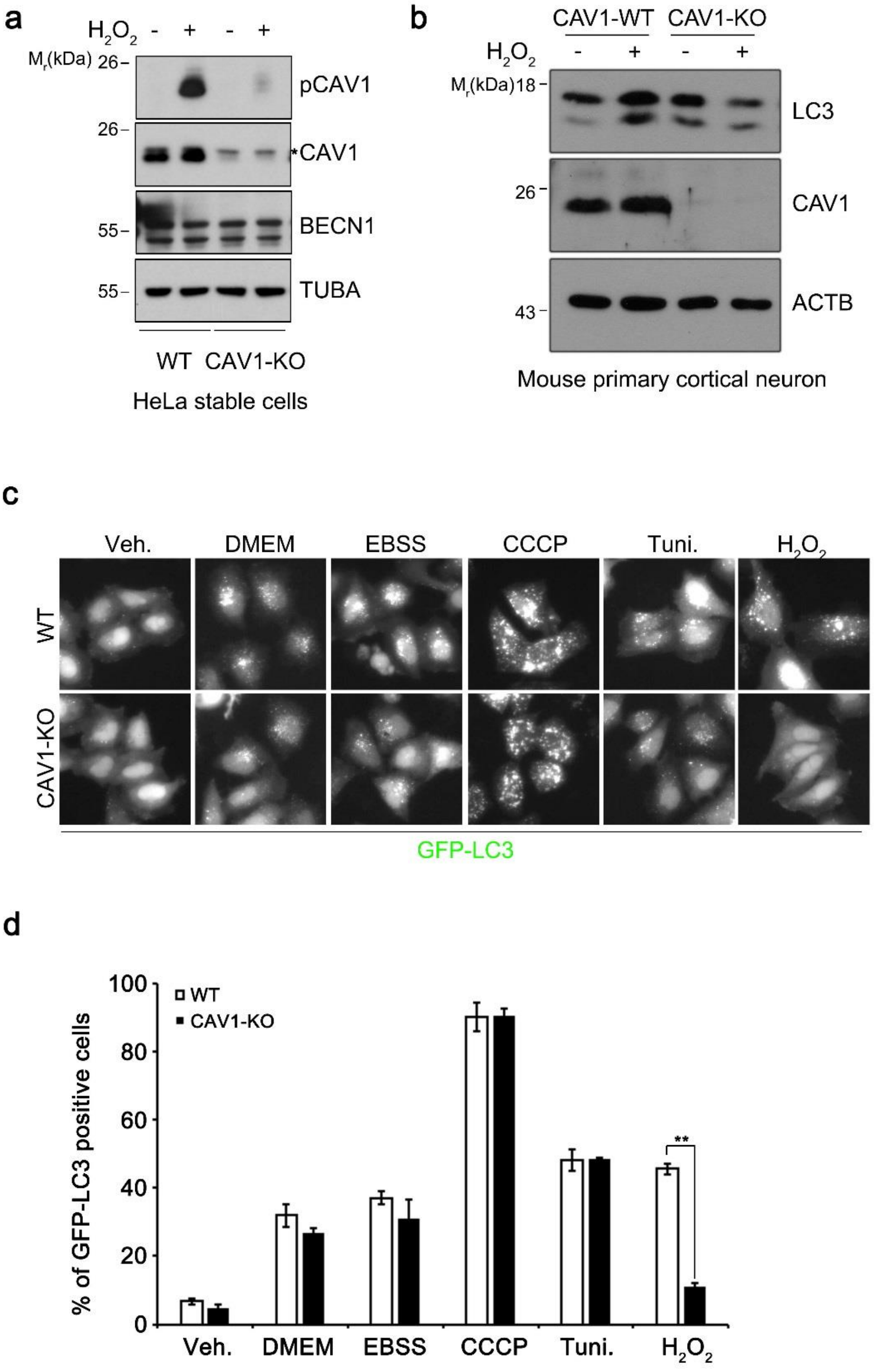

Supplementary Figure 2. Nah et al.

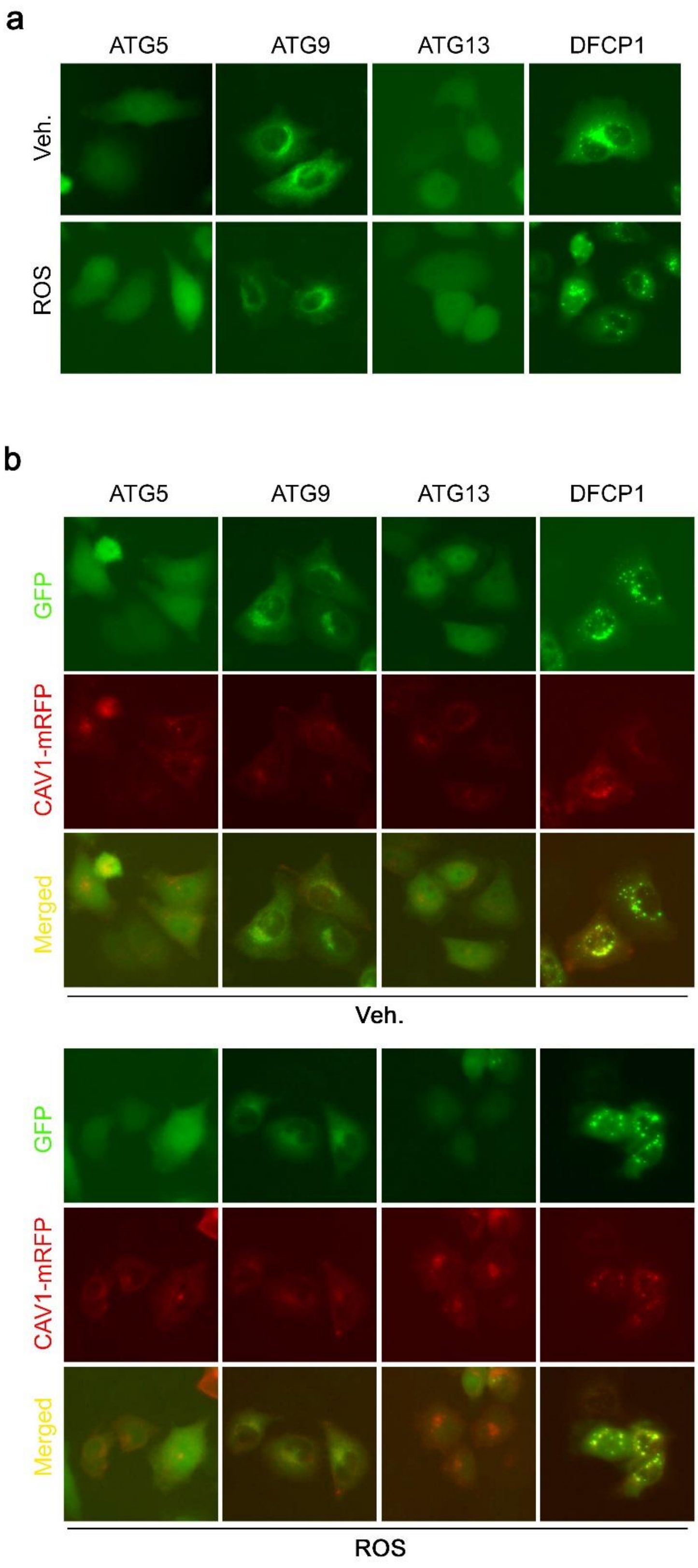

Supplementary Figure 3. Nah et al.

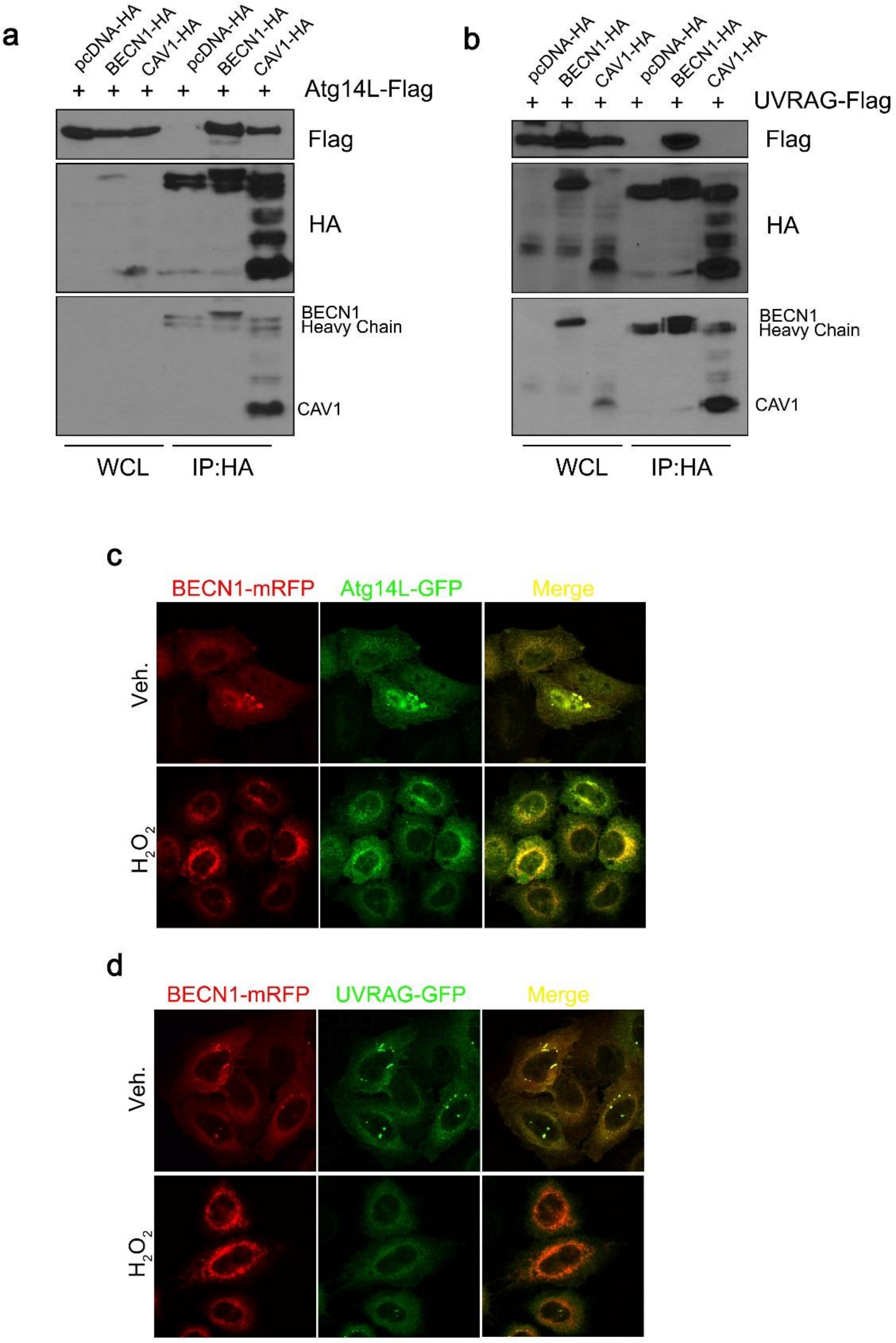

Supplementary Figure 4. Nah et al.

a

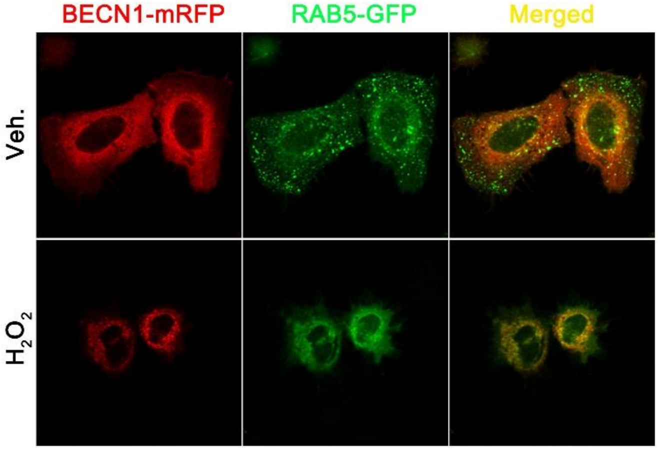

b

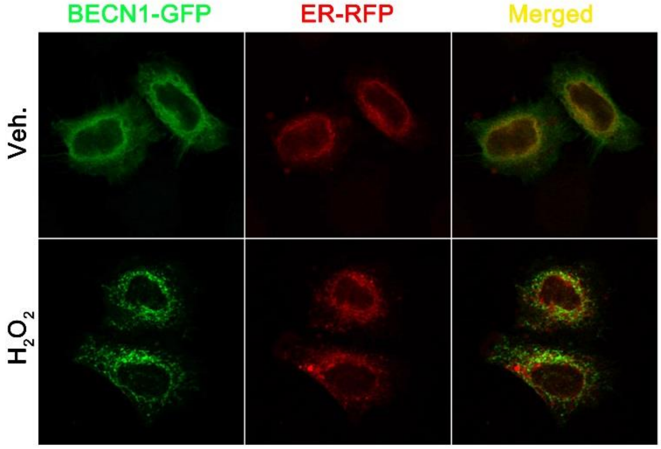

Supplementary Figure 5. Nah et al.

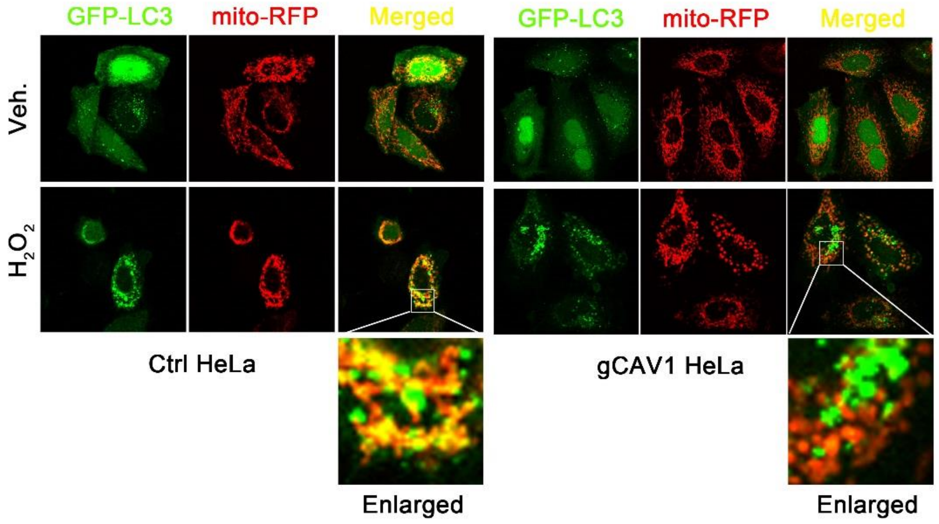

Supplementary Figure 6. Nah et al.

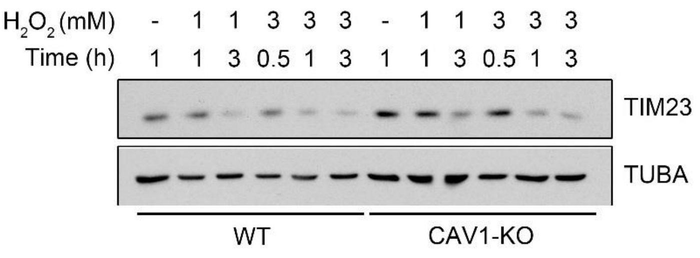

Supplementary Figure 7. Nah et al.

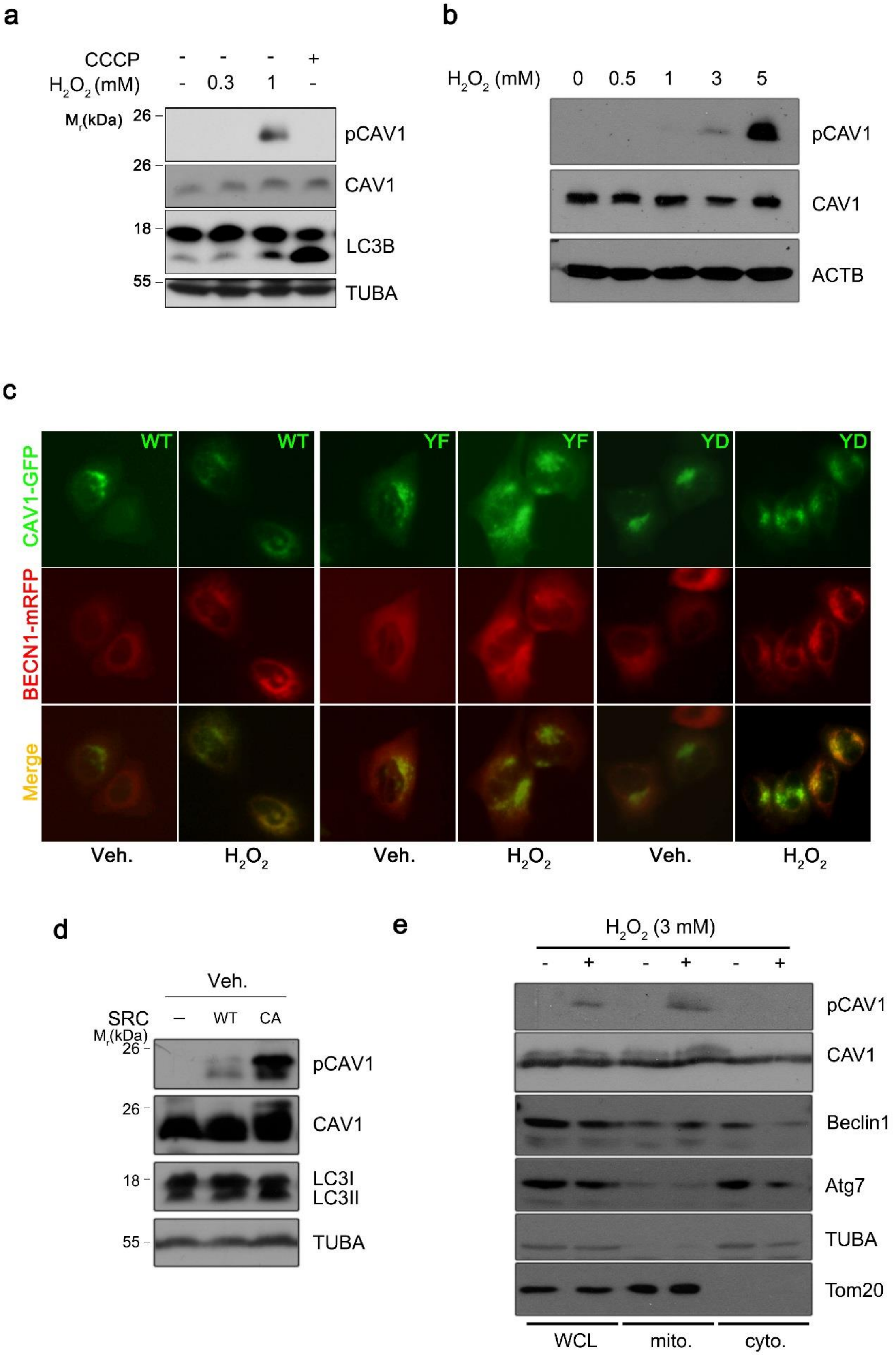

Supplementary Figure 8. Nah et al.

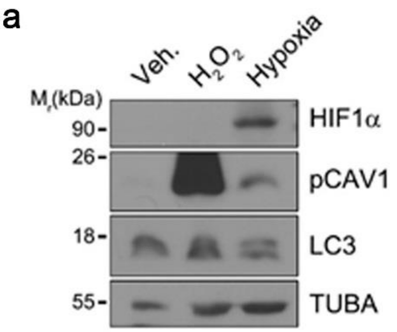

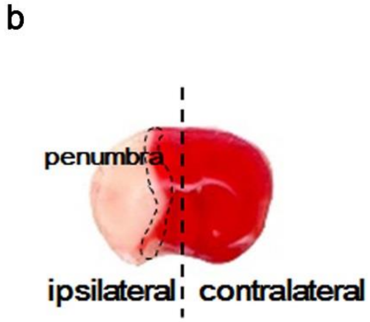

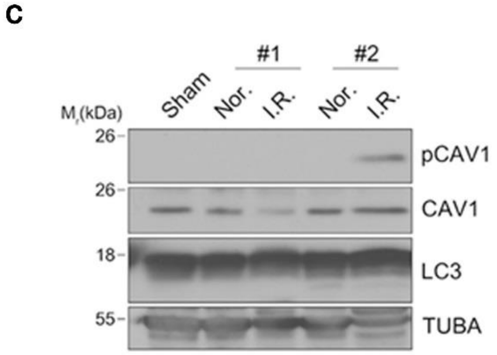

Supplementary Figure 9. Nah et al.

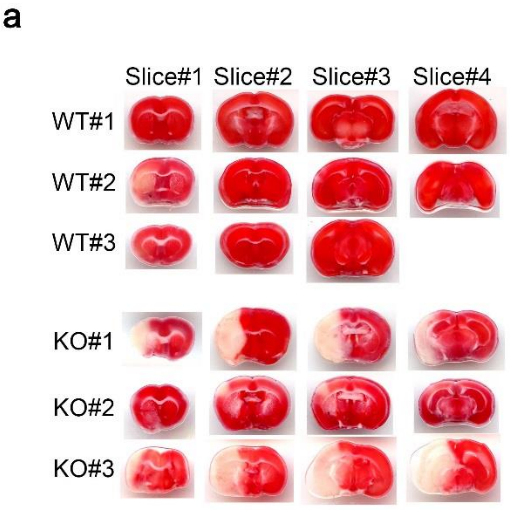

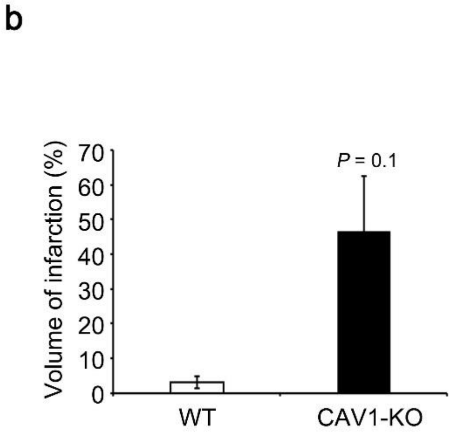

Supplementary Figure 10. Nah et al.

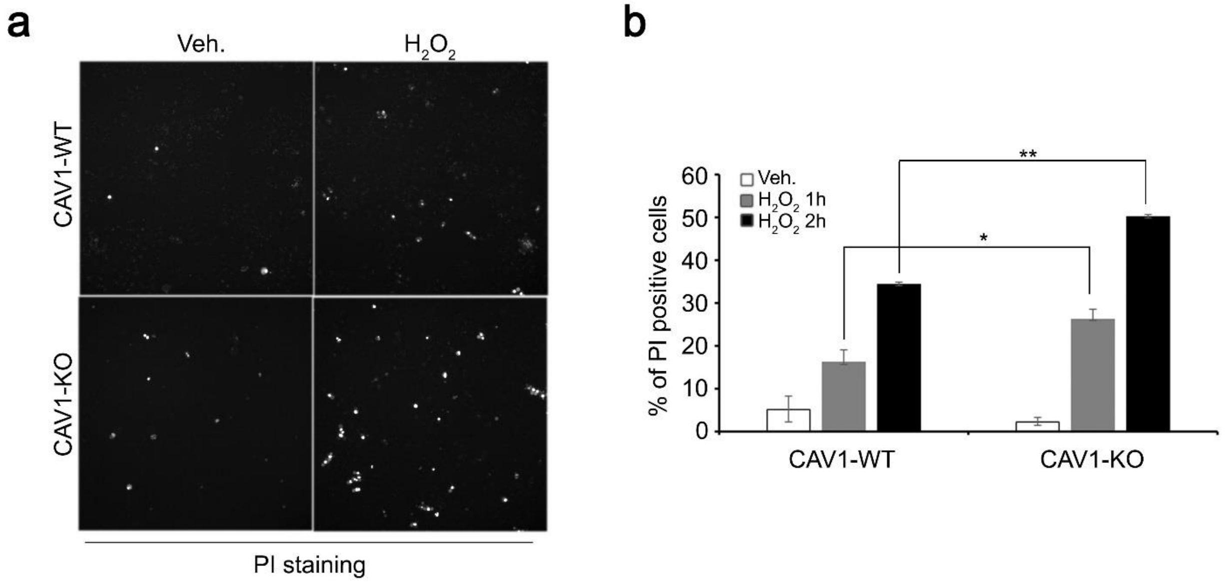

Supplementary Figure 11. Nah et al.

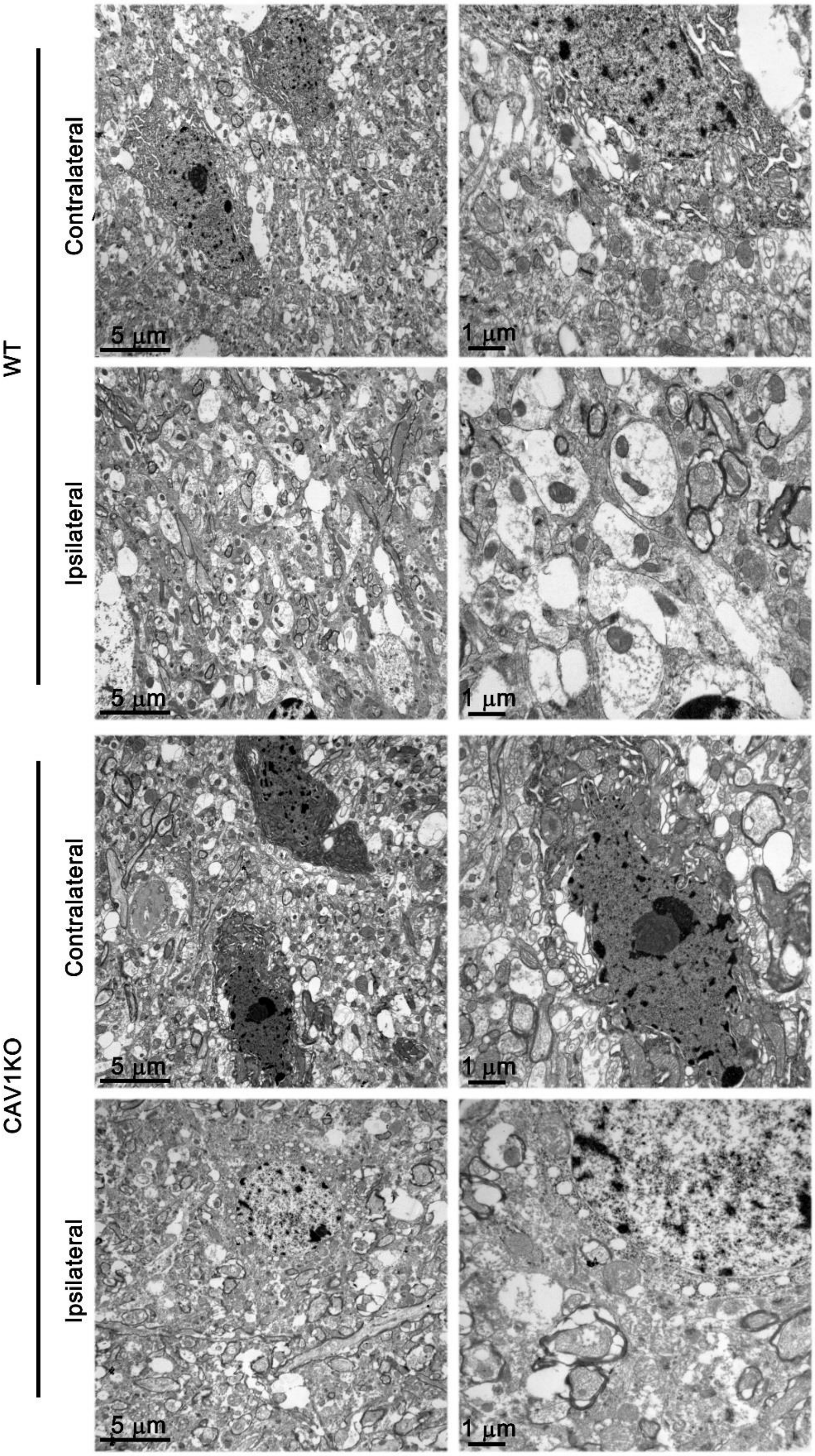

Supplement: Supplementary Figures [file cddis201771x2.pdf]
